# Maternal Nutrient Restriction Programs Fetal Hepatic DNA Methylation in Ovine Monozygotic Twins

**DOI:** 10.3390/ijms27031553

**Published:** 2026-02-04

**Authors:** Megan E. Miller, Emilie C. Baker, Michael C. Satterfield

**Affiliations:** 1 Department of Animal Science, Texas A&M University, College Station, TX 77843, USA; megelizmill1@tamu.edu; 2 Department of Agricultural Sciences, West Texas A&M University, Canyon, TX 79016, USA; ebaker@wtamu.edu

**Keywords:** WGBS, DNA methylation, fetal liver, maternal nutrition, metabolism, sheep

## Abstract

Maternal nutrient restriction (MNR) heightens disease susceptibility in offspring through epigenetic modifications that alter the development of essential organs. This study investigates how restriction alters the fetal sheep hepatic methylome and its potential regulatory influence on gene expression. Using a monozygotic twin model generated through embryo splitting, we examined hepatic DNA methylation responses to maternal nutrient restriction (50% vs. 100% NRC nutritional requirements; *n* = 4 per group) from gestational day (GD) 35 to 135 in pregnant sheep. At GD 135, conceptus (fetal–placental unit) development was assessed; although fetal weight was unaffected (*p* > 0.10), restricted fetuses exhibited reduced liver mass (*p* < 0.05). Whole-genome bisulfite sequencing (WGBS) of fetal liver identified 1,636,305 differentially methylated CpG sites (dmCpGs) in the Group-Level Analyses and 42,231 dmCpGs in the Twin-Pair Analyses. At the Group-Level, 40,533 promoter, 126,667 exonic, and 785,381 intronic sites were identified, whereas the Twin-Pair subset contained 1314, 7116, and 22,239, respectively. Site-level shifts and functional enrichment across features highlighted GPCR–cAMP/calcium–PI3K/AKT signaling, phosphoinositide metabolism, ECM/integrin–focal adhesion networks, thyroid hormone signaling, and Rho-family GTPases. These findings indicate that maternal nutrient restriction modifies the fetal hepatic methylome through coordinated signaling, metabolic, and structural reconfigurations that create conditions conducive to metabolic disease.

## 1. Introduction

Maternal nutrition during pregnancy is a critical determinant of fetal development and lifelong health. Deviation from optimal nutrition disrupts placental efficiency, fetal growth, and postnatal metabolic regulation across species, including livestock and humans [1,2,3,4]. Foundational epidemiological evidence from the Dutch Famine Winter, along with subsequent large-scale cohort analyses, demonstrated that maternal undernutrition increases offspring risk for metabolic and cardiovascular disorders in adulthood [5,6,7,8,9,10]. These observations underpin the Developmental Origins of Health and Disease framework, which posits that adverse intrauterine environmental cues during critical developmental windows can durably alter organ trajectories and increase disease susceptibility, especially under prenatal–postnatal nutritional mismatch [11].

Among affected organs, the fetal liver is especially vulnerable given its position as the first organ to receive nutrients entering the fetal circulation from the placenta, coupled with its rapid developmental remodeling to establish hematopoietic and metabolic programs [12,13,14,15,16,17,18,19]. Supporting this notion, meta-analytic evidence in sheep identifies the fetal liver as the organ showing the most consistent reductions in absolute mass during gestation and postnatal development [20]. Furthermore, because maternal nutrition can be readily manipulated in sheep during gestational windows that closely align with key periods of human fetal organogenesis, sheep serve as a relevant translational model for human developmental programming [21].

Nutrient restriction during key periods of gestation has been linked to persistent transcriptional and physiological changes in hepatic function [22,23,24]. Early restriction has been shown to alter liver growth and modulate growth-factor and apoptotic signaling [25,26], with even brief insults affecting liver size [27,28]. Mid-gestational restriction produces sex-specific shifts in hepatic gluconeogenic enzymes and metabolic gene expression [29,30]. These shifts may arise as downstream consequences of perturbed placental function and glucocorticoid handling, as evidenced by reduced placentome mass, diminished 11β-HSD2 activity [21], and recalibrated fetal HPA responsiveness [31]. Late-gestation maternal nutrient restriction impairs hepatic structure and metabolic maturation, reducing liver mass, protein and lipid content, and increasing collagen deposition [32], thereby disrupting the architectural organization essential for hepatic function [14,33,34]. Even in instances where liver size normalizes at birth, offspring exhibit glucose intolerance, insulin resistance, and increased adiposity later in life [35]. Severe late-gestation restriction further triggers profound metabolic disturbances, including elevated fatty acid oxidation and suppressed lipid and sterol biosynthesis, which are linked to altered glycerophospholipid pathways and impaired cell-cycle progression [36]. Despite these well-documented phenotypic and metabolic consequences, the understanding of the molecular basis driving these outcomes remains incomplete.

Epigenetic mechanisms offer a compelling explanation for how gestational insults become biologically embedded as lasting modifications in gene regulation and phenotype [6,37]. DNA methylation, in particular, is a stable and heritable mark that integrates environmental input with transcriptional regulation and is widely recognized as a central mechanism linking maternal nutrient status to fetal programming [38,39,40]. Studies in rodents and humans demonstrate that prenatal nutrition alters methylation at genes regulating lipid and carbohydrate metabolism, endocrine signaling pathways, and neuroendocrine appetite circuits [3,41]. Although much of this work has focused on promoters, methylation across the broader genomic landscape also carries regulatory significance. Emerging evidence indicates that exonic methylation contributes to regulatory complexity via alternative splicing, while first-intron methylation exhibits an inverse association with gene expression across tissues and species [5,39,42,43,44].

Despite substantial evidence that maternal nutrient restriction alters metabolic function [25,26,29,30,32,36,45] and contributes to nutrition-sensitive epigenetic remodeling across diverse tissues [2,3,40,46,47,48], two major gaps remain. First, most studies employ genetically diverse populations, making it difficult to disentangle environmental effects from genetic variability. Second, limited attention has been given to regional methylation patterns, which may reveal distinct biological outputs. Furthermore, variation in experimental design, especially in the timing and duration of restriction, complicates comparisons and obscures whether observed effects reflect gestational stage, genetic background or treatment responses. These limitations collectively restrict mechanistic interpretation and underscore the need for approaches capable of isolating environmental inputs with higher resolution.

With this background, the present study offers a key methodological strength to investigate the effects of maternal nutrient restriction on fetal hepatic methylation by leveraging a monozygotic twin-pair design that controls for genetic background and enhances sensitivity to environmentally driven epigenetic effects. Embryos derived from a single zygote were split and transferred into separate recipient female sheep fed either 100% (CON) or 50% (RES) of National Research Council (NRC) nutrient requirements from GD 35 to 135 [49]. Utilizing whole-genome bisulfite sequencing (WGBS), DNA methylation at CpG sites was first profiled for treatment-level comparison (Group-Level Analysis) and then extended to the twin-pair level (Twin-Pair Analysis), to capture robust, reproducible methylation responses distinct from pair-specific or biologically inherent variation. This two-tiered framework was further stratified across promoter, exonic, and intronic regions to delineate region-specific regulatory roles. Both analytical tiers characterized the magnitude, directionality, and spatial organization of feature-specific differentially methylated CpG sites and were subjected to region-stratified functional interpretation using KEGG, Reactome, and Gene Ontology (GO) enrichment analyses. To our knowledge, this combined approach has not been previously applied to fetal liver WGBS under MNR.

## 2. Results

### 2.1. Phenotypic Effect on Organs Due to Maternal Nutrient Restriction at GD 135

Maternal nutrient restriction (RES, 50% NRC) did not change fetal body weight, placentome weight, or placentome number compared with controls (CON, 100% NRC) at GD 135. However, the liver, spleen, pancreas, and small intestine weights were reduced in the RES fetuses. All other organs and tissues outlined in Table 1 were not different (*p* > 0.05) between groups.

### 2.2. Distribution of DNA Methylation in the Fetal Hepatic Genome

High-quality whole-genome bisulfite sequencing datasets were obtained for downstream analyses (Table S1). Differential methylation was then summarized across promoter, exon, and intron regions using two complementary statistical approaches (Table S2). The Group-Level Analysis, which compared fetuses gestated in the 50% nutrient group to those in the 100% group (padj ≤ 0.05; |methDiff| ≥ 0.10), identified 1,636,305 total dmCpGs (Table 2). Regionally, 40,533 sites localized to promoter regions, 126,667 to exonic regions, and 785,381 to intronic regions, with the remaining sites located in intergenic regions. Because individual CpG sites may be co-annotated to more than one genomic feature, regional counts are non-exclusive and will not sum to the genome-wide total. Across all regions, differential methylation was strongly biased toward hypermethylation in nutrient-restricted fetuses relative to controls. Site-to-gene annotation yielded 4795 promoter-associated genes, 9156 exon-associated genes, and 11,318 intron-associated genes, providing the basis for functional enrichment analyses.

When refined to the Twin-Pair Analysis criterion, 42,231 total dmCpGs were identified (Table 3). To derive this set, pairwise files were filtered using the same statistical thresholds applied to the Group-Level file (Table S2), and sites shared by at least three of the four pairs were retained. Regionally, 1314 sites localized to promoters, 7116 to exons, and 22,239 to introns. Mirroring the Group-Level results, hypermethylation in nutrient-restricted fetuses predominated across all genomic features relative to controls. Feature-specific dmCpGs were visualized by chromosome and methylation difference for both analyses (Figures S1 and S2). Dashed lines denote the ±0.10 methDiff threshold used to define dmCpGs. Across genomic features, dmCpGs were evenly dispersed across the entire genome rather than clustered by chromosome.

Further analysis of the Twin-Pair WGBS data revealed a severity-dependent response across pairs. Pairs exhibiting greater liver mass loss displayed higher numbers of dmCpGs and a stronger imbalance toward hyper-methylation relative to hypomethylation (Table S2). Within-pair liver-to-body mass ratios (liver g/kg) were used to calculate a Liver Sparing Index (LSI), where values < 1 indicate relative liver loss and values > 1 indicate relative liver sparing (Table S3). Using this metric, 3 of 4 pairs showed relative liver loss (LSI = 0.791, 0.820, 0.900), whereas one pair showed relative liver sparing (LSI = 1.048). Correspondingly, liver g/kg was reduced in RES offspring from pairs exhibiting relative liver loss but elevated in the liver-spared RES offspring. This severity-dependent pattern is unlikely to reflect technical variation, given the uniformly high WGBS metrics.

### 2.3. Differential DNA Methylation Within Promoter Regions Between CON and RES Fetal Liver

Based on statistical analyses, 40,533 promoter-dmCpGs were identified at the Group Level, while 1314 promoter-dmCpGs were identified under stricter Twin-Pair criterion. Using absolute methylation difference, the top 30 genes were identified for each analysis (Table 4 and Table 5). Comparison of the top 30 promoter-region dmCpGs from the Group-Level and Twin-Pair analyses revealed three shared sites annotating to MROH9, MB21D2, and C18H15orf40. While these sites represent the most reproducible promoter methylation events, their corresponding genes remain minimally characterized in hepatic contexts. Accordingly, evaluation was expanded beyond the rank-limited top 30 to examine the full set of promoter-associated dmCpGs meeting statistical thresholds. This broader view revealed clear multi-CpG clusters associated with GNAS, RXRA, and EHMT1. This observation is consistent with evidence that promoter methylation typically functions as spatially correlated blocks or differentially methylated regions that relate more robustly to transcriptional regulation than single isolated CpGs, warranting focused interpretation [44].

**Figure 1:**
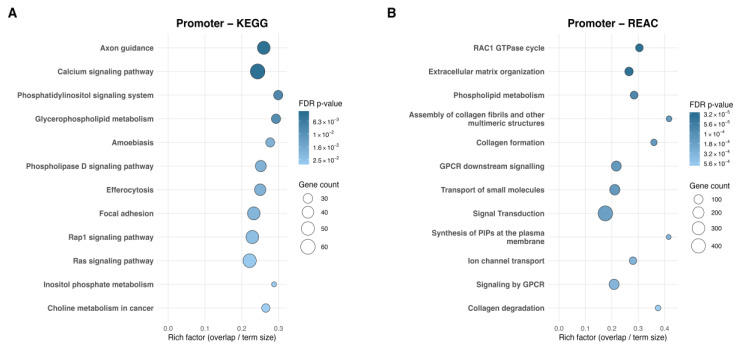
Group-Level Scatterplots of pathways enriched for promoter-associated dmCpGs analyzed by KEGG and Reactome. (**A**) KEGG analysis of genes associated with Group-Level promoter dmCpGs and (**B**) Reactome analysis of genes associated with Group-Level promoter dmCpGs, showing the top 12 significantly enriched pathways per database. The *x*-axis represents the rich factor (overlap/term size), and the *y*-axis lists pathway names. Point size reflects the number of dmCpG-associated genes, and color indicates the adjusted *p*-value (FDR q ≤ 0.05).

Moving from site-level signals to pathway-level integration highlighted how dmCpGs organize into broader biological pathways. Group-Level functional enrichment identified 25 KEGG and 68 Reactome pathways (FDR q ≤ 0.05), of which the top 12 pathways per database are shown in Figure 1A,B. KEGG enrichment highlights axon guidance, calcium signaling, phosphatidylinositol signaling, and glycerophospholipid metabolism, with additional pathways involving phospholipase D, Rap1, and Ras signaling cascades. Focal adhesion and inositol phosphate metabolism further reflect structural and metabolic themes, respectively. Reactome enrichment complements these themes, revealing phospholipid metabolism, structural pathways including extracellular matrix organization and collagen assembly, formation, and degradation, as well as signaling modules such as the RAC1 GTPase cycle, signal transduction, and GPCR-mediated pathways.

**Figure 2:**
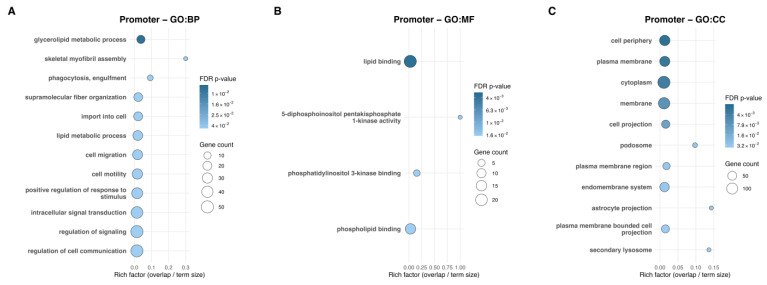
Twin-Pair Gene Ontology enrichment of promoter-associated dmCpGs. (**A**) GO Biological Process (GO:BP), (**B**) GO Molecular Function (GO:MF), and (**C**) GO Cellular Component (GO:CC) terms enriched among genes associated with Twin-Pair promoter dmCpGs. Across GO categories, 17 GO:BP, 4 GO:MF, and 11 GO:CC terms met significance, with the only top 12 GO:BP terms shown. The *x*-axis displays the rich factor (overlap/term size), and the *y*-axis lists the enriched terms. Point size reflects the number of dmCpG-associated genes, and point color reflects the adjusted *p*-value (FDR q ≤ 0.05).

Twin-Pair functional enrichment did not yield significant KEGG or Reactome pathways. However, Twin-Pair promoter-level Gene Ontology analysis revealed distinct enrichment patterns across functional categories. In total, 17 Biological Process (GO:BP) terms reached statistical significance, of which the top 12 are displayed in Figure 2A, while only 4 Molecular Function (GO:MF) terms (Figure 2B) and 11 Cellular Component (GO:CC) terms (Figure 2C) met significance thresholds. More specifically, GO:BP identified glycerolipid metabolic process as the top enriched term. Consistent with this, GO:MF showed tightly coordinated enrichment across four lipid-associated functions—lipid binding, 5-diphosphoinositol pentakisphosphate 1-kinase activity, phosphatidylinositol 3-kinase binding, and phospholipid binding. In parallel, GO:CC enrichment localized these functional changes to membrane-associated regions, including the cell periphery, plasma membrane, cytoplasm, membrane, and cell projection. These patterns indicated that promoter-level dmCpGs converge on lipid-related functions and membrane-associated cellular domains. Gene Ontology for the Group-Level promoter analysis is provided in Supplementary Figure S3A–C, displaying the top 12 significantly enriched Biological Process, Molecular Function, and Cellular Component terms (FDR q ≤ 0.05).

### 2.4. Differential DNA Methylation Within Exonic Regions Between CON and RES Fetal Liver

Exon-associated dmCpGs totaled 126,667 in the Group-Level dataset and 7116 in the Twin-Pair dataset following statistical analyses. The top 30 sites in each analysis were reported based on absolute methylation difference (Table 6 and Table 7). Although no site-level overlap was observed between analyses, both datasets demonstrated top-ranked sites central to hepatic metabolic integration and structural organization. The Group-Level analysis highlighted dmCpGs within metabolic and signaling regulators such as PIK3C2G, FGFBP3, and PTPRJ, whereas the Twin-Pair dataset identified three notable CpG sites within MCUB. Additional dmCpGs outside the top-ranked lists revealed remodeling within ECM and cytoskeletal loci (DMD, SHROOM2, RELN).

**Figure 3:**
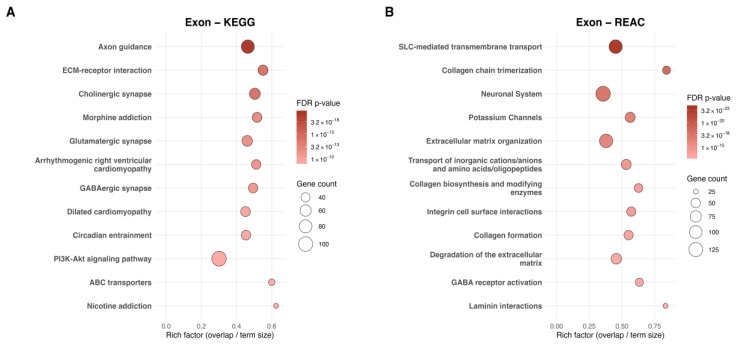
Group-Level Scatterplots of pathways enriched for exon-associated dmCpGs analyzed by KEGG and Reactome. (**A**) KEGG analysis of genes associated with Group-Level exonic dmCpGs and (**B**) Reactome analysis of genes associated with Group-Level exonic dmCpGs, showing the top 12 significantly enriched pathways per database. The *x*-axis represents the rich factor (overlap/term size), and the *y*-axis lists pathway names. Point size reflects the number of dmCpG-associated genes, and color indicates the adjusted *p*-value (FDR q ≤ 0.05).

Exonic site-level patterns were contextualized using functional enrichment across analytical tiers. Group-Level enrichment identified 125 KEGG and 220 Reactome pathways (Figure 3A,B). Group-level KEGG results emphasized axon guidance, ECM–receptor interaction, and PI3K–AKT signaling, whereas Reactome captured ECM, collagen, and laminin interactions, along with potassium channel pathways. Neuronal and synaptic signaling, as well as diverse transport mechanisms, appeared in both panels.

**Figure 4:**
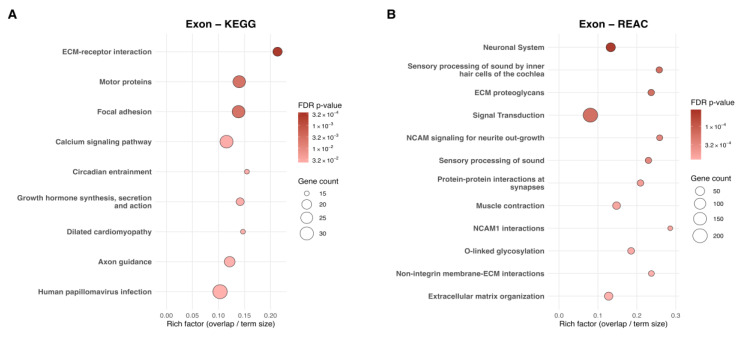
Twin-Pair Scatterplots of pathways enriched for exon-associated dmCpGs identified by KEGG and Reactome analyses. (**A**) KEGG analysis of genes associated with twin-pair exonic dmCpGs, for which nine pathways met significance, and (**B**) Reactome analysis of genes associated with twin-pair exonic dmCpGs, showing the top 12 significantly enriched pathways. The *x*-axis represents the rich factor (overlap/term size), and the *y*-axis lists pathway names. Point size reflects the number of dmCpG-associated genes, and color indicates the adjusted *p*-value (FDR q ≤ 0.05).

Twin-pair enrichment identified 9 KEGG and 64 Reactome pathways (Figure 4A,B), where core ECM and cytoskeletal themes were retained; however, transporter pathways were not preserved. Instead, KEGG results showed new emergence of calcium signaling and growth hormone synthesis, secretion, and action, while Reactome shifted toward more specific neuronal, NCAM-mediated, and ECM–proteoglycan processes. Exon-associated Gene Ontology is provided for Group-Level analyses in Supplementary Figure S3D–F and for Twin-Pair analyses in Supplementary Figure S4A–C.

### 2.5. Differential DNA Methylation Within Intronic Regions Between CON and RES Fetal Liver

Intronic methylation changes were widespread, with 785,381 total dmCpGs detected in the Group-Level comparison and 22,239 total dmCpGs identified in the Twin-Pair analysis. Absolute magnitude of methylation difference was used to rank the top 30 sites (Table 8 and Table 9). Similar to the exonic region, there was no site-overlap between analyses, yet the metabolic and structural themes observed in earlier enrichments were clearly extended into intron-specific regulatory signatures. Group-Level dmCpGs mapped to metabolic regulators such as PLAAT1 and ADCY9, as well as intracellular signaling components including PTPRD and RASGEF1B. Additional remodeling was evident in structural loci (LTBP1, ROBO1) and stress-responsive genes (ATF6, UVRAG). Twin-Pair filtering refined these patterns toward nutrient-sensing and energy-prioritizing regulators such as WDR59 and SOS1.

**Figure 5:**
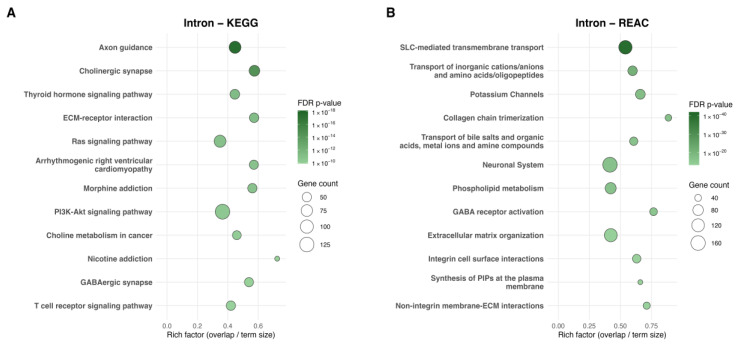
Group-Level Scatterplots of pathways enriched for intron-associated dmCpGs analyzed by KEGG and Reactome. (**A**) KEGG analysis of genes associated with group-level intronic dmCpGs and (**B**) Reactome analysis of genes associated with Group-Level intronic dmCpGs, showing the top 12 significantly enriched pathways. The *x*-axis represents the rich factor (overlap/term size), and the *y*-axis lists pathway names. Point size reflects the number of dmCpG-associated genes, and color indicates the adjusted *p*-value (FDR q ≤ 0.05).

Functional enrichment analyses further contextualized these findings. Group-Level enrichment revealed 192 KEGG and 393 Reactome pathways (Figure 5A,B). Intron-associated KEGG terms included axon guidance, cholinergic synapse, and thyroid hormone signaling, along with pathways shared across promoter and exon analyses such as ECM–receptor interaction, Ras signaling, and PI3K–AKT signaling. Reactome enrichment highlighted transmembrane transport and ion channel mechanisms, with cross-region convergence in ECM-, collagen-, and integrin-related processes, neuronal system pathways, and metabolic themes including phospholipid metabolism and plasma membrane PIP synthesis.

**Figure 6:**
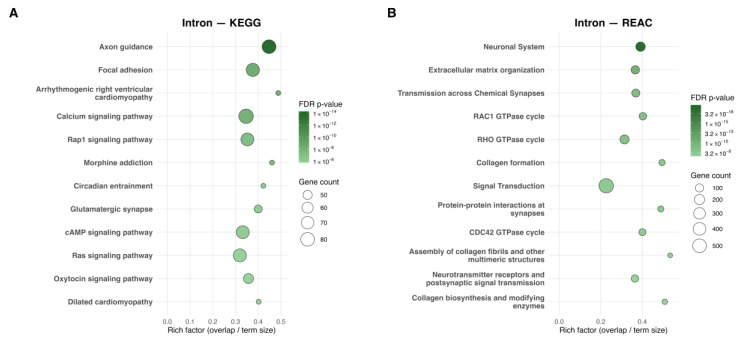
Twin-Pair Scatterplots of pathways enriched for intron-associated dmCpGs identified by KEGG and Reactome analyses. (**A**) KEGG analysis of genes associated with twin-pair intronic dmCpGs and (**B**) Reactome analysis of genes associated with twin-pair intronic dmCpGs, showing the top 12 significantly enriched pathways. The *x*-axis represents the rich factor (overlap/term size), and the *y*-axis lists pathway names. Point size reflects the number of dmCpG-associated genes, and color indicates the adjusted *p*-value (FDR q ≤ 0.05).

Twin-Pair enrichment identified 94 KEGG and 133 Reactome pathways (Figure 6A,B). Axon guidance and Ras signaling were retained, while calcium and cAMP signaling were newly enriched. Reactome results emphasized highly specific RAC1, RHO, and CDC42 GTPase cycles, alongside continued enrichment of neuronal systems, collagen formation, and broader ECM organization. Intron-associated Gene Ontology is provided for Group-Level analyses in Supplementary Figure S3G–I and for Twin-Pair analyses in Supplementary Figure S4D–F.

## 3. Discussion

Maternal nutrient restriction during pregnancy has long been associated with impaired placental development, reduced placental efficiency, and restricted fetal growth across ruminant models [50]. However, neither placental size nor fetal weight fully explain metabolic risk prevalence; instead, organ-specific developmental changes often provide more insight into how MNR reshapes tissue-specific regulatory programs with downstream metabolic consequences. Across fetal programming studies, growth impairments are not uniform across fetal tissues, with the liver consistently experiencing perturbations in growth patterns in response to maternal undernutrition [20,46,51,52,53,54]. These reductions align with altered hepatic architecture, metabolic zonation, and mRNA expression that influence liver function across the lifespan [25,55,56]. While recent studies have begun to examine hepatic DNA methylation in nutritionally influenced metabolic disease contexts, the genomic resolution and regional specificity of MNR-induced methylation remain incompletely characterized, and interpretation is further complicated by the strong influence of genetic heterogeneity seen in previous work [46]. To address this gap, our monozygotic twin-pair design provides a genetically controlled framework to isolate maternal diet effects on individual regions of the fetal hepatic methylome.

### 3.1. Impact of MNR on Monozygotic Offspring

Previous studies in our laboratory using this same nutritional model have consistently demonstrated classic IUGR outcomes, including reductions in placental, fetal, and organ weights [57,58,59,60,61]. Unlike these studies, the present study’s objective was to specifically conduct epigenetic analyses using a novel, technically challenging monozygotic twin model with limited availability of twin-pairs. Therefore, it is not surprising, nor concerning, that statistical significance was not met for parameters such as placental and fetal weights while still showing significance for individual organ weights, such as the liver.

### 3.2. Genome-Wide Hypermethylation and dmCpG Burden Under MNR

Across tiers, WGBS identified a genome-wide, region-consistent increase in DNA methylation in response to MNR. Although this pattern was consistent across analyses, its magnitude varied at the twin-pair level, with greater liver mass loss showing higher numbers of dmCpGs and a stronger imbalance in hyper- versus hypomethylated sites across genomic regions (Table S1). From a developmental perspective, the observed bias toward hypermethylation could be interpreted as a maladaptive feature, in which increased methylation constrains proliferative or growth-associated gene programs, contributing directly to reduced liver mass and altered structural organization. Alternatively, within a metabolic programming framework, these same hypermethylation patterns may arise secondarily as part of an adaptive response to a smaller liver under nutrient-limited conditions. Here, genome-wide hypermethylation may serve to limit transcriptional plasticity and energetically expensive developmental networks. Under this interpretation, hypermethylation may not be inherently maladaptive, but instead reflects context-dependent epigenetic remodeling that attempts to balance reduced developmental capacity with postnatal metabolic function.

An alternative, and not mutually exclusive, explanation is that hypermethylation arises from impaired epigenetic remodeling activity during fetal liver development. Modest but consistent site-level evidence within TET1 at both promoter and intronic sites under each analyses could reflect reduced demethylation capacity, thereby permitting accumulation of methylated CpGs through diminished 5mC turnover [62]. Additional cross-tiered intragenic hypermethylation within TET2 and TET3 suggests further suppression across the TET-family with potential transcriptional influence [39,43]. Under such conditions, DNMT1-mediated maintenance methylation stabilizes these marks during cell division, progressively reinforcing a hypermethylated state [62]. Although these patterns support a plausible mechanism whereby impaired demethylation contributes to genome-wide hypermethylation, definitive molecular drivers remain to be established and will require targeted functional investigation. Nonetheless, the present findings indicate that widespread hypermethylation is a defining epigenetic feature of MNR in the ovine fetal liver, with its magnitude proportional to the degree of liver mass loss.

### 3.3. Promoter dmCpGs Highlight Nutrient- and Hormone-Sensitive Regulatory Hubs

Promoter methylation is a core regulator of transcriptional output and remains one of the most widely used epigenetic indicators of gene expression potential [63,64]. Accordingly, promoter-associated dmCpGs may provide the most direct insight into regulatory perturbations experienced by RES offspring. One of the most notable promoter-level remodeling events involved an imprinted gene, GNAS, which displayed a broad 47.5 kb promoter-proximal methylation domain in the Group-Level analysis that refined to a 45.8 kb region containing four CpGs under the Twin-Pair design (chr13:57,173,598–57,219,371). Although additional imprinted genes were observed across the tiered datasets in this study, the present analysis focuses on GNAS as a representative example, given its role in encoding the stimulatory G-protein alpha subunit (Gsα), a key mediator of GPCR-driven cAMP production [65]. In hepatic contexts, Gsα knockout in mice decreases gluconeogenesis, increases glycogen synthesis, enhances insulin sensitivity in other tissues, and increases circulating glucagon and GLP-1 levels [66]. Separately, GNAS knockdown in human hepatocellular carcinoma cells inhibits LPS induced-IL-6 expression by attenuating STAT3 activation [65]. Consequently, GNAS promoter hypermethylation in RES offspring suggests a coordinated dampening of hepatic cAMP signaling and altered STAT-related responsiveness, a mechanistic pattern that recurs across exonic and intronic dmCpGs in our study.

RXRA showed a remodeled 44.6 kb domain spanning its promoter-proximal and first intronic elements that refined to a single reproducible CpG (chr6:1,892,179). Modulation at this locus is of interest given RXRA’s essential role as the heterodimeric partner of PPARA, a complex that governs fatty-acid oxidation and ketogenesis. Human studies reinforce the nutrient-sensitive nature of this locus, demonstrating that low maternal carbohydrate intake in early pregnancy is associated with increased RXRA promoter methylation in umbilical cord tissue, which in turn predicts higher child body mass index and fat mass later in life [67]. Consistent with these observations, mice with hepatocyte-specific RXRA deficiency (hRXR⍺ ko) exhibit elevated serum triglyceride concentrations and increased hepatic apoCIII mRNA expression, a key inhibitor of triglyceride clearance [68]. These findings indicate that RXRA is an essential regulatory component of fatty-acid homeostasis and that its disruption may predispose restricted offspring to dyslipidemia.

EHMT1 (GLP) presented a highly compact and internally consistent promoter signature, with a 1058 bp hypermethylated block refining to an exceptionally tight 63 bp window (chr3:367,230–367,293) that encompassed six dmCpGs shared across three of four twin pairs, all exhibiting concordant hypermethylation with highly consistent methylation differences across sites. Notably, the liver-sparing pair was the only pair lacking promoter-level changes at this locus. Despite minimal characterization of EHMT1 within the liver, evidence from extrahepatic systems implicates its role in metabolic regulation. Adipose-specific loss of EHMT1 disrupts thermogenesis and insulin sensitivity, while clinical features of Kleefstra syndrome further link EHMT1 to early-life metabolic dysregulation, with both contexts frequently accompanied by obesity [69,70]. Consistent with these observations, increased adiposity has also been reported in *Ehmt2 (G9a)* and *Ehmt1* knockout mice [69]. Biochemically, EHMT1 forms an obligate heterodimer with EHMT2, constituting the predominant H3K9me1/2 methyltransferase system [71], suggesting that disruption of either component may have downstream consequences. While hepatic functions of EHMT1 remain poorly defined, the metabolic effects of EHMT2 loss have been more extensively characterized. *Ehmt2* knock-out has been shown to induce fatty liver, even in the absence of detectable hepatic deletion, in addition to being associated with increased food intake and significant upregulation of adipogenic markers (*PPARγ*, *C/EBPα*, and *aP2*). Additionally, EHMT2 deficiency reduces hepatic H3K9me1/2 levels and perturbs lipid and insulin signaling pathways, including disruption of HMGA1, a key regulator of INSR transcription [69,72]. In this context, the present study’s identification of EHMT1 promoter hypermethylation alongside intronic-hypermethylation of HMGA1 in the Twin-Pair dataset therefore introduces a fetal-specific regulatory pattern not previously described in the liver. These findings raise the possibility that altered EHMT1 methylation may influence EHMT1/EHMT2–HMGA1 axes during a period when hepatic chromatin states are highly plastic. Such alterations may have postnatal consequences for insulin-responsive and lipid-handling pathways, although these effects remain to be confirmed.

### 3.4. Promoter-Level Functional Enrichment Reveals Coordinated Membrane and Signaling Network Remodeling

Promoter-level functional enrichment revealed a strong mechanistic convergence across KEGG, Reactome, and Gene Ontology. At the Group-Level, promoter-associated dmCpGs mapped to coordinated networks governing membrane-associated metabolism and membrane-initiated signaling. Although the Twin-Pair promoter dataset contained too few genes to meet statistical thresholds for KEGG or Reactome enrichment, coherent GO:BP, GO:MF, and GO:CC terms recapitulated these biological themes. Enriched pathways included glycerophospholipid and phospholipid metabolism, phosphoinositide turnover, and GPCR-, Rap1-, and Ras-linked signaling, all of which are well documented in metabolically stressed liver. To contextualize these findings, we first considered evidence for altered hepatocyte membrane lipid metabolism, followed by its consequences for membrane-initiated signaling networks. Across human and mouse models of metabolic-dysfunction-associated steatotic liver disease (MASLD), integrated multi-omics analyses reveal depletion of major membrane glycerophospholipids (PC, PE, and CL) coincides with increased glycerolipid (TG) accumulation and impaired insulin signaling, reflecting coordinated disruption of phospholipid-dependent membrane and phosphoinositide signaling pathways [73]. Notably, unlike adult MASLD where lipid excess drives triglyceride accumulation, IUGR sheep experience impaired lipid oxidation and storage, resulting in free fatty acid accumulation and lipotoxic stress in the absence of overt steatosis [74]. Regardless of triglyceride accumulation or depletion, nutritional stress disrupts hepatocyte lipid buffering, predisposing the liver to free fatty acid-driven membrane instability and metabolic dysfunction.

Consistent with altered membrane lipid metabolism, disrupted GPCR crosstalk in metabolic liver disease impairs phosphoinositide turnover and PLC/IP_3_–Ca^2+^ signaling, aligning with phospholipid and PIP-cycle pathways enriched in the same Reactome panel. KEGG enrichment further reinforces membrane-initiated signaling themes, highlighting Ras and Rap1 as complementary axes. Aberrant Ras activation has been linked to disruptions in hepatic lipid metabolism, altering triglyceride handling and fatty-acid regulatory programs [75]. Rap1 similarly modulates hepatocyte lipid metabolism and insulin responsiveness, with loss of Rap1 activity promoting steatosis and selective insulin resistance in obese mice liver [76]. Downstream of these membrane-initiated signaling pathways, rat nutrient-restriction models demonstrate altered mTOR activity and dysregulated SREBP1-driven lipogenesis, reflecting how nutrient-sensitive signaling cascades translate membrane perturbations into transcriptional and metabolic outcomes [77]. Together, promoter-associated functional enrichment reveals coordinated remodeling of hepatocyte membrane function through altered nutrient-sensing circuitry, with implications for downstream metabolic regulation.

### 3.5. Exonic dmCpGs Implicate Insulin, FGF, and Calcium-Dependent Metabolic Signaling

Given that exon methylation can influence transcriptional elongation, splicing decisions, and local chromatin structure, the exonic dataset offers interpretable regulatory signals distinct from promoter and intron associated effects [39,78,79]. At the Group-Level, a dmCpG within PIK3C2G (chr3:197,427,220) emerged as a central node associated with nutrient sensing. As a Class II PI3K, PIK3C2G generates PI(3,4)P_2_ within endosomal membranes, sustaining AKT2 activation following insulin stimulation. Mouse models lacking PIK3C2G show depletion of this endosomal PI(3,4)P_2_ pool, leading to selective loss of AKT2 phosphorylation, reduced glycogen synthase activity, and diminished hepatic glycogen storage [80]. These defects manifest as hyperlipidemia, adiposity, and progressive insulin resistance, particularly under metabolic challenge. Thus, hypermethylation of PIK3C2G in RES fetal livers may reflect altered regulatory dynamics, with potential consequences for AKT2-dependent anabolic tone and hepatic glycogen deposition.

Complementing this architecture, a dmCpG within FGFBP3 (chr22:13,243,347)—one of the strongest and most notable hypomethylated loci across the Group-Level dataset—reinforces emerging disruptions in hepatic carbohydrate and lipid metabolism. FGFBP3 enhances the biological activity of endocrine FGFs, particularly FGF19 and FGF21, which modulate glucose handling and lipid balance through AKT and STAT3 signaling [81]. Loss of FGFBP3 has been shown to impair glucose clearance and elevate circulating lipids, whereas FGFBP3 overexpression improves glucose regulation and reduces hepatic expression of lipogenic enzymes (*FASN*, *SREBP1c*, *ACC1*) [81]. Although exonic methylation has not been definitively linked to expression outcomes, the presence of a strong hypomethylated CpG within FGFBP3 may serve as a compensatory mechanism in RES offspring, whereby blunted IL-6/STAT3 activation inferred from promoter hypermethylation at GNAS could increase reliance on FGF-mediated signaling to sustain metabolic responsiveness. In the same manner, hypermethylation of PTPRJ (chr15:76,786,768), a negative regulator of FGFR and PI3K–AKT cascades, may reflect a reduced ability to temper excessive FGF-driven activation [82]. This interpretation remains speculative but highlights FGFBP3 and PTPRJ as relevant loci within the exonic landscape.

A similar interpretive framework applies to MCUB (chr6:16,095,414–16,095,430), which emerged as a notable dmCpG in the Twin-Pair dataset. MCUB encodes a regulatory subunit of the mitochondrial calcium uniporter (MCU) complex that restricts mitochondrial Ca^2+^ influx to prevent oxidative injury. Dysregulation of MCU-dependent Ca^2+^ signaling in hepatocytes has been increasingly linked to obesity, type 2 diabetes mellitus, and metabolic-associated fatty liver disease [83]. Moreover, recent work in murine models of metabolic dysfunction-associated steatohepatitis (MASH) demonstrates that MCU knockdown alleviates steatosis and fibrosis by downregulating lipogenic enzymes and enhancing PPARγ signaling [83]. Accordingly, reproducible differential methylation within MCUB during fetal development may increase susceptibility to hepatic metabolic dysfunction. However, functional validation is required to establish causality.

### 3.6. Exon-Level Functional Enrichment Reveals ECM-Driven Mechanotransduction and Cytoskeletal Remodeling

Exon-level functional enrichment revealed a distinct, structurally oriented architecture compared with promoter enrichment and broadened the interpretive scope beyond what was evident from exonic site-level data alone. Top pathways—including ECM–receptor interaction, collagen-associated processes, and focal adhesion signaling—are central to mechanotransduction at the plasma membrane, where extracellular mechanical cues are converted into intracellular biochemical outputs [84]. Upon integrin clustering, phosphoinositide 3-kinases (PI3K), phospholipase C, and associated signaling molecules can be recruited to focal adhesions, enabling force-dependent signal propagation [84]. Therefore, these structural enrichments at the plasma membrane may contribute to the PI3K signaling observed across both exonic and intronic Group-Level KEGG analyses. In metabolic disease contexts, increased hepatic expression of ECM proteins is associated with insulin resistance, with integrin signaling proposed as a key mechanistic intermediary linking matrix remodeling to impaired insulin action [85].

Twin-Pair enrichment expanded this architecture by revealing calcium signaling and growth hormone (GH) synthesis, secretion, and action as prominent exonic pathways. Evidence from murine liver models demonstrate that disturbances in lipid and calcium homeostasis can impair endoplasmic reticulum stability and contribute to broader metabolic dysfunction [86]. When considered alongside differential methylation of MCUB, these findings suggest that MNR influences early Ca^2+^-dependent regulatory systems. The appearance of GH activity likely reflects its role in cytoskeletal regulation, rather than its well-known metabolic functions, given the surrounding enrichment of ECM organization, focal adhesion, and motor protein pathways. In this context, GH promotes actin rearrangement, microtubule polymerization, and the assembly of adhesion-related multiprotein complexes that govern cell movement and morphology [87]. At the same time, matrix–integrin interactions can also modulate growth factor and IGF-family receptor activity, indicating a bidirectional relationship within developing hepatic tissue [88]. Collectively, exon enrichment sheds light on how structural and mechanical properties of the fetal liver microenvironment influence hepatic signaling pathways that govern metabolic efficiency.

### 3.7. Intronic dmCpGs Implicate Metabolic, Hormone-Sensitive, and Growth-Regulatory Signals

Introns represent an important regulatory layer in gene expression, with functional effects that depend on intronic position relative to transcriptional control elements. Methylation within the first intron is strongly associated with transcriptional regulation and is often inversely correlated with gene expression [43], while promoter-proximal introns can enhance transcriptional efficiency in mammalian cells [89]. In contrast, methylation in downstream introns appears to influence gene output primarily through effects on alternative splicing, mRNA accumulation, and transcript structure rather than transcriptional initiation alone [39,90]. Notably, intron-mediated regulation can occur independently of intron removal and may involve splice-site-associated sequences and cis-acting regulatory elements within full-length introns [89]. Together, these findings suggest that while intron 1 has a well-established transcriptional role, downstream introns constitute a distinct and underexplored regulatory layer in gene expression.

Among Group-Level intron dmCpGs, a site at chr1:194,992,857 in PLAAT1, located in the first intron in several PLAAT1 transcript isoforms (for example, XM_060404656.1), emerged as a particularly informative locus linking membrane lipid remodeling to downstream metabolic capacity. Experimental models demonstrate that PLAAT1 deficiency alleviates high-fat diet-induced obesity and hepatic steatosis, accompanied by downregulation of key lipogenic enzymes (*ACC1*, *FASN*) and transcriptional regulators (*SREBP1c*, *PPARγ*), alongside enhanced insulin-dependent AKT phosphorylation [91]. Conversely, PLAAT1 overexpression promotes mitochondrial fragmentation and peroxisome loss in an enzyme activity-dependent manner, implicating this pathway in organelle stress and lipid dysregulation [92]. Hypermethylation within the first intron of PLAAT1 may therefore reflect reduced transcriptional output, aligning with the broader suppression of AKT–PPAR–SREBP signaling observed at the promoter level, while simultaneously implicating constrained mitochondrial capacity at the exon level. Although such repression may be adaptive in utero, postnatal downregulation could compromise lipid turnover, fatty acid oxidation flexibility, and long-term hepatic insulin responsiveness.

A second major dmCpG was located within ADCY9 (chr24:3,910,367). Although isoform-specific evidence for ADCY9 remains limited, adenylate cyclases collectively serve as critical regulators of GPCR- and calcium-mediated cAMP production—a signaling axis highly enriched at the promoter level. Group-Level analyses revealed widespread modulation across the adenylate cyclase family (ADCY1–10), which refined under Twin-Pair filtering to a subset of isoforms (ADCY2/3/5/6/8). Among these, ADCY8 and ADCY3 were particularly notable, as elevated ADCY8 activity has been associated with obesity and type 2 diabetes, while ADCY3 deficiency increases susceptibility to diet-induced obesity [93]. Furthermore, the dmCpG within PTPRD (chr2:76,906,769) identified a metabolically relevant locus with established suppressive roles in metabolic liver disease [94]. PTPRD encodes a protein tyrosine phosphatase that modulates STAT3 and PI3K–AKT signaling—pathways previously implicated at the promoter level—positioning it as a key regulatory brake on hepatic glucose and lipid metabolic pathways [94,95,96]. Reduced inhibitory control at this locus may facilitate short-term metabolic adaptation under nutrient scarcity, while embedding susceptibility to insulin resistance and fibrotic remodeling through dysregulated STAT3 signaling later in life. Finally, additional remodeling at structural genes such as COL11A1 (chr1:42,191,480) and LTBP1 (chr11:41,299,778) further reinforce concurrent modulation of matrix-related processes observed across exonic and intronic layers [97,98].

Under the Twin-Pair analysis, dmCpGs within WDR59 and SOS1 were identified. Intronic hypermethylation of WDR59, a regulatory component of the GATOR2 complex, suggests recalibration of mTORC1 sensitivity under nutrient-restricted conditions. Given that gestational undernutrition suppresses GATOR2-mediated mTORC1 activation, methylation-based attenuation at this locus is consistent with reduced anabolic signaling and a shift toward metabolic conservation in RES fetuses [99]. Concurrent hypermethylation of SOS1, the primary Ras activator, implicates altered extracellular matrix regulation. Loss of SOS1 has been shown to increase collagen I expression (via PI3K–AKT signaling), elevate total collagen protein, slightly increase fibronectin expression, and alter fibroblast proliferation and migration through specific downstream pathways [100]. This interpretation aligns with the extensive collagen and fibronectin remodeling observed across the full Twin-Pair intronic site-level dataset.

### 3.8. Intron-Level Functional Enrichment Reveals Hormone-Sensitive and Morphogenic Network Integration

Intron-level functional enrichment recapitulated many of the themes observed at the promoter and exonic levels, including ECM, collagen, focal adhesion, integrin-mediated networks, Ras/Rap1, calcium, PI3K–Akt signaling, phospholipid metabolism, and PIP synthesis, while also revealing additional regulatory dimensions. The emergence of thyroid hormone (TH) signaling is particularly notable when conjoined with the metabolic themes previously identified. Recent experimental evidence supports this framework by demonstrating that T3 reshapes hepatocyte metabolic programming through direct chromatin engagement, with TRβ1 and ChREBP co-occupying lipogenic genes to regulate lipid synthesis while TRβ1 separately controls fatty acid oxidation [101]. This dual action mirrors pathways repeatedly enriched across genomic regions in our analysis. In the same manner, the new appearance of cAMP signaling within the Twin-Pair KEGG results aligns with the numerous nutrient and hormone-responsive pathways previously identified as cAMP dependent. Finally, the presence of small GTPase-linked elements (RHOA, RAC1, and CDC42) in the Twin-Pair Reactome results adds breadth to the structural and mechanotransduction themes observed across regions [102]. Disruption of CDC42, specifically, has been shown to reduce hepatoblast protrusions and alter liver budding dynamics, implicating early perturbations in hepatogenesis [103]. In this context, intronic small GTPase enrichments, coupled with ECM-associated exonic enrichment, suggest that MNR modulates the structural and mechanical development of the fetal liver.

### 3.9. Cross-Regional Convergence in DNA Methylation Within the Sheep Fetal Liver

**Figure 7:**
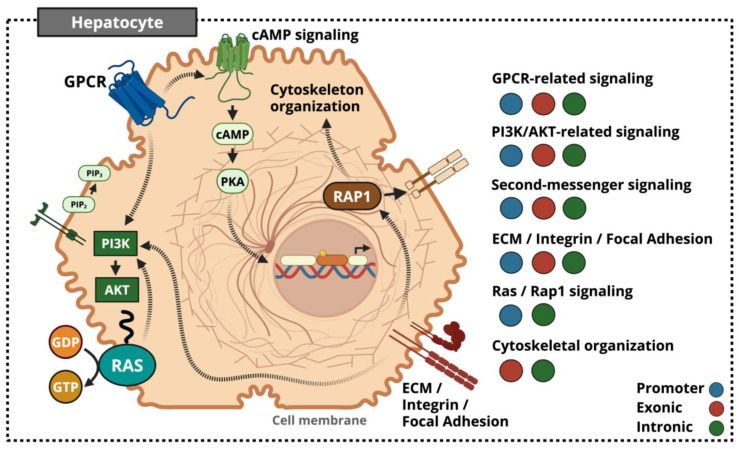
Integrated model of cross-regional DNA methylation convergence in the fetal sheep hepatocyte following maternal nutrient restriction. Promoter-, exon-, and intron-associated dmCpGs collectively implicate membrane-initiated signaling (GPCR-related, PI3K–AKT-related, Second-messenger, and Ras/Rap1 pathways) alongside ECM, focal adhesion, and cytoskeletal organization. Colored circles denote genomic regions in which site-level methylation or pathway enrichment was observed. Created in BioRender. Miller, M. (2026) https://BioRender.com/boa040z (accessed on 28 January 2026).

While promoter, exon, and intron methylation were analyzed separately to preserve regional specificity, integration across these layers revealed convergent biological pathways governing hepatocyte signaling, membrane dynamics, and structural organization (Figure 7). Rather than acting in isolation, differential methylation across genomic features consistently targeted pathways involved in membrane-initiated nutrient and hormone sensing, including GPCR–cAMP signaling, PI3K–AKT signaling, calcium-dependent second-messenger systems, and small GTPase networks such as Ras and Rap1. Promoter-associated dmCpGs implicated altered GPCR signaling capacity and phospholipid–phosphoinositide metabolism, while exonic and intronic methylation further reinforced modulation of PI3K–AKT and Ras/Rap1-mediated signal propagation. In parallel, recurrent enrichment of ECM, integrin, focal adhesion, and cytoskeletal pathways across all genomic regions highlights coordinated regulation of mechanotransduction and structural integrity within the developing hepatic microenvironment.

Together, these cross-regional methylation patterns indicate coordinated epigenetic remodeling of membrane-initiated signaling and hepatocyte structural organization. Such integration suggests that maternal nutrient restriction reshapes hepatocyte architecture in ways that alter how these cells interpret and transmit metabolic, hormonal, and mechanical cues during fetal development. While these changes may represent adaptive strategies that prioritize cellular stability and metabolic conservation in utero, the same structural and signaling configurations are likely to become maladaptive when postnatal nutrient environments diverge from those anticipated.

## 4. Materials and Methods

### 4.1. Experimental Design

Mature Hampshire ewes of similar parity, frame size, and initial body-condition score (BCS) were superovulated and bred by natural service on day 0 to one of three half-brother rams. As part of a large-scale embryo transfer protocol, embryos were recovered on day 6 at the compacted morula to blastocyst stage, bisected, and each demi-embryo was transferred to a separate synchronized, multiparous Hampshire recipient ewe of comparable BCS to generate monozygotic twins gestating in different mothers; embryo transfer techniques have been published previously [58]. Pregnancy was diagnosed by ultrasound on Day 28. Recipient ewe pairs in which both halves of the split embryo established a viable pregnancy were retained for this study, where they were individually housed in pens with concrete flooring and provided a 7-day diet transition period. Recipient ewes in which only one demi-embryo established a pregnancy were assigned to a separate project. On day 35 of pregnancy, retained recipient ewes were assigned randomly to either a control-fed group, CON (100% NRC; *n* = 4), or a nutrient-restricted group, RES (50% NRC; *n* = 4); composition of their respective diets has been published previously [61]. This model induced a total caloric restriction equally across macromolecule groups in the RES ewes until GD 135, while keeping vitamins and minerals provided as recommended or in excess for all ewes [104]. Ewes were fed once daily, body weight was measured weekly, and feed intake was adjusted based on changes in body weight. This paired-recipient approach resulted in genetically identical fetuses developing simultaneously, but separately, in ewes exposed to different dietary treatments. On GD 135, ewes were humanely euthanasia and necropsies were performed as previously described [57]. Full-uterus weight, placentome number and weight, amniotic and allantoic fluid volumes, and fetal weight were recorded. All fetal organs were weighed, and tissues were collected for subsequent analyses. All experimental procedures in this study were approved by, and performed in accordance with, the Texas A&M University Institutional Animal Care and Use Committee (AUP#2015-0204) and the National Institutes of Health (NIH) guidelines.

### 4.2. Liver Tissue Processing, DNA Extraction, and WGBS Library Preparation

Liver tissues were collected from the central region of the right lobe and then finely minced to ensure representation of all major hepatic cell populations, before being snap-frozen in liquid nitrogen and stored at −80 °C. Samples were shipped on dry ice to Zymo Research Corp. (Irvine, CA, USA), where DNA extraction and whole-genome bisulfite sequencing were performed, following the manufacturer’s instructions. DNA extraction was conducted using the Quick-DNA Miniprep Plus Kit (Zymo Research, Irvine, CA, USA). Genomic DNA (100 ng per sample) underwent bisulfite conversion using the widely adopted EZ DNA Methylation-Lightning Kit (Zymo Research, Irvine, CA, USA; Cat. D5031) [105,106,107,108,109]. After conversion, a second-strand synthesis reaction was performed, and adapters were incorporated via tagmentation using Illumina’s Nextera^®^ kit (Illumina, San Diego, CA, USA). PCR was then performed with Illumina Nextera Unique Dual Indices, and library quality control was conducted on the Agilent 4200 TapeStation (Agilent, Santa Clara, CA, USA). Finally, libraries were sequenced on an Illumina NovaSeq X platform (150 bp PE reads, Illumina, San Diego, CA, USA).

### 4.3. Basic Bioinformatics Analyses

Sequence reads from WGBS libraries were identified using Illumina Real-Time Analysis (RTA) software (v4.29.2). Raw FASTQ files were adapter and quality trimmed, and 15 bases were further trimmed off at the 5′ end according to the Nextera recommendations using TrimGalore. FastQC was used to assess the effect of trimming and overall quality distributions of the data. Picard tools were used to calculate the library insert size distribution. To process WGBS data, trimmed reads were aligned to the ovine reference genome assembly ARS-UI_Ramb_v3.0 (GCF_016772045.2) using Bismark (v0.24.2). Methylation ratios for each cytosine in CpG context were called using MethylDackel (v0.6.1). The methylation level of each sampled cytosine was estimated as the number of reads reporting a C, divided by the total number of reads reporting a C or T. Read depths per cytosine in the genome were calculated and tabulated using scripts.

### 4.4. Differential Methylation Analyses and Annotation

Differentially methylated CpG sites were identified using the DSS framework (v2.46.0; R version: 4.2.3). The data was pre-filtered to exclude low coverage cytosines and keep cytosines with read depth ≥ 5 in ≥2 samples in any group. DSS performed Wald testing with Benjamini–Hochberg correction, and statistically significant sites were identified at FDR ≤ 0.05. Methylation difference (methDiff) was calculated as the mean methylation in the 50% NRC group minus the mean methylation in the 100% NRC group for group-level comparisons, while pairwise methylation difference was calculated as methylation in the 50% NRC twin minus the matched 100% NRC twin. Positive methDiff values indicate hypermethylation, defined as higher methylation in the 50% NRC (RES) group or offspring relative to the 100% NRC (CON) control, whereas negative methDiff values indicate hypomethylation relative to controls. Annotation of dmCpGs was performed using reference data from NCBI and the UCSC Genome Browser by overlapping each dmCpG with functional genomic features, including genes, exons, introns, promoters, and CpG islands. This annotation yielded NCBI RefSeq accession numbers. The minimum size for an overlap was 1 base pair. QC metrics and download links for the raw and processed sequencing files were obtained from the custom MultiQC report generated by Zymo Research.

### 4.5. Methylation Analyses Strategy

DSS-derived methylation calls generated by Zymo Research were subsequently processed using our tiered filtering pipeline in RStudio (version 2025.05.0+496). The Group-Level analyses applied the thresholds padj ≤ 0.05 and |methDiff| ≥ 0.10 to identify all significant dmCpGs. The Twin-Pair analyses applied the same statistical thresholds as the Group-Level analyses to each pairwise comparison and additionally required sites to be shared across at least three of the four twin pairs to be retained. The rationale for performing both analyses is to ensure robustness of epigenomic inference, thereby mitigating false positives and emphasizing reproducibility.

### 4.6. Pathway and Enrichment Analyses

To interpret the functional relevance of dmCpG sites, pathway and gene-ontology (GO) enrichment analyses were performed on feature-specific gene sets following threshold filtering and transcript-to-gene mapping. RefSeq transcript accessions (XM/XR/NM/NR; version suffixes removed) were converted to gene symbols using NCBI E-utilities via the R package rentrez (an Entrez API key was used only to increase request throughput). Only records with NCBI:txid9940 were retained. Transcripts unresolved by NCBI were queried in Ensembl BioMart release 114, dataset ARS-UI_Ramb_v2.0, using the external-reference filters RefSeq mRNA ID(s), RefSeq mRNA predicted ID(s), RefSeq ncRNA ID(s), and RefSeq ncRNA predicted ID(s). Entries were then collapsed by gene symbol to generate unique gene lists for enrichment. Enrichment was performed with g:Profiler (R package gprofiler2 v0.2.3) using the gost function (organism = hsapiens), querying KEGG, Reactome, and GO:BP/MF/CC simultaneously. Multiple testing was controlled by Benjamini–Hochberg FDR (correction_method = “fdr”). Unless otherwise noted, terms were considered significant at FDR q ≤ 0.05. The g:Profiler default annotated gene universe was used as background, and term-to-gene intersections were returned (evcodes = TRUE) for downstream visualization. For figures, each (source × region) result was summarized as a bubble plot showing the top terms ranked by FDR (ties broken by enrichment magnitude). Bubble size encodes the intersection size (genes from our list annotated to the term), bubble fill encodes −log10(FDR), and the *x*-axis shows the rich factor (intersection size/term size). Promoter, exon, and intron results were visualized in consistently colored panels and plotted as separate KEGG and Reactome figures for each genomic region and analytical tier using ggplot2 and patchwork in RStudio. The same plotting framework was used to generate GO enrichment panels, which are provided in the Supplementary Materials.

## 5. Conclusions

Maternal nutrient restriction induced multi-layer remodeling of promoter, exon, and intron DNA methylation, thereby rewiring how the fetal liver integrates extracellular signals with intracellular mechanics. The strongest and most reproducible shifts converged on GPCR and PI3K/AKT signaling cascades, phospholipid and membrane turn-over/activity, and extracellular matrix and cytoskeletal stability. Together, these pathways capture a core fetal-programming strategy in which the hepatic methylome recalibrates cellular structure and signaling to maintain metabolic stability under conditions of limited substrate availability. Through selective redistribution of DNA methylation, the fetal liver appears to optimize survival in a nutrient-restricted intrauterine environment. Importantly, the extent of this redistribution appears to vary in accordance with intrinsic biological adaptability to nutrient restriction among genetically identical offspring. Nevertheless, when postnatal conditions diverge from those for which these adaptations were established, the same epigenetic configurations may predispose offspring to metabolic dysfunction and related health vulnerabilities.

While this study provides high-resolution evidence of coordinated epigenomic remodeling, direct causal relationships between DNA methylation and transcriptional outcomes cannot be inferred from the present data. Accordingly, future studies should incorporate targeted, candidate-gene validation using qPCR to assess locus-specific expression changes associated with the methylation patterns identified here. In addition, allele-resolved methylation analyses will be necessary to define parental allele specificity and to clarify the extent to which imprinting-related regulatory mechanisms contribute to fetal hepatic programming under maternal nutrient restriction.

## Figures and Tables

**Table 1:** Placental Metrics and Fetal Organ/Tissue Weights at GD135 under MNR.

	CON (*n* = 4) 100% NRC	RES (*n* = 4) 50% NRC	SEM ^2^	*p*-Value
Panel A: Placental Measurements and Fetal Weight				
Placentome Number	86	76	4	0.252
Placentome Weight (g)	547	564	48	0.871
Fetal Weight (kg)	5.603	4.956	0.241	0.200
Panel B: Fetal Organ and Tissue Weights ^1^				
Brain	50.1	53.4	1.1	0.140
Left Ventricle	11.7	11.1	0.6	0.612
Right Ventricle	14.2	13.3	0.8	0.633
Septum	10.1	9.0	0.4	0.158
Thymus	10.8	8.1	1.1	0.245
Spleen	7.5	5.7	0.4	0.007
Adrenals	0.490	0.485	0.045	0.960
Lungs	206	186	7	0.198
Liver	152	119	8	0.015
Pancreas	4.46	3.80	0.17	0.031
Small Intestine	185	139	11	0.021
Kidneys	26.4	22.8	1.3	0.112
Gastrocnemius Muscle	12.0	11.1	0.6	0.520
Soleus Muscle	4.92	4.59	0.20	0.461
L. Dorsi Muscle	44.8	41.6	2.0	0.459
Brown Adipose Tissue	14.5	15.9	0.8	0.430
Omental Fat	3.31	3.26	0.20	0.918

^1^ Fetal organ and tissue weights are expressed in grams (g). ^2^ SEM: standard error of the mean.

**Table 2:** Distribution of dmCpG sites across genomic regions in the Group-Level Analysis.

Genomic Region	Site Count ^b^	Proportion ^c^	Associated Genes	Hypermethylated Sites ^d^	Hypomethylated Sites ^e^
Total	1,636,305	1.00	—	1,614,260	22,045
Promoter	40,533	0.03	4795	39,357	1176
Exon	126,667	0.08	9156	123,595	3072
Intron	785,381	0.48	11,318	773,239	12,142
Genic ^a^	830,632	0.51	—	817,357	13,275
Intergenic	783,214	0.48	—	774,985	8229

^a^ Genic denotes both exon and intron regions; due to co-annotation, exon and intron counts will not sum to genic total. ^b^ Site counts across genomic features will not equal the total as sites can be assigned multiple annotation categories. ^c^ Proportions may not sum to 1.00 due to co-annotation. ^d^ Hypermethylated sites correspond to positive methDiff values (50% NRC > 100% NRC). ^e^ Hypomethylated sites correspond to negative methDiff values (50% NRC < 100% NRC).

**Table 3:** Distribution of dmCpG sites across genomic regions in the Twin-Pair Analysis.

Genomic Region	Site Count ^b^	Proportion ^c^	Mapped Genes	Hypermethylated Sites ^d^	Hypomethylated Sites ^e^	Mixed Sites ^f^
Total	42,231	1.00	—	39,849	2228	154
Promoter	1314	0.03	417	1136	178	0
Exon	7116	0.17	1680	6369	682	65
Intron	22,239	0.53	4313	20,980	1203	56
Genic ^a^	24,942	0.59	—	23,360	1478	104
Intergenic	16,657	0.39	—	15,944	663	50

^a^ Genic denotes both exon and intron regions; due to co-annotation, exon and intron counts will not sum to genic total. ^b^ Site counts across genomic features will not equal the total as CpG sites can be assigned multiple annotation categories. ^c^ Proportions may not sum to 1.00 due to co-annotation. ^d^ Hypermethylated sites correspond to positive methDiff values (50% NRC > 100% NRC). ^e^ Hypomethylated sites correspond to negative methDiff values (50% NRC < 100% NRC). ^f^ Concordant directionality was not enforced for sites meeting the ≥3-of-4 pair criterion.

**Table 4:** Group-Level Top 30 dmCpG Sites across Promoter Regions Ranked by Absolute methDiff.

OAA ^a^	Position	Difference ^b^	Adjusted *p*-Value	Gene
X	16,384,928	0.5176	3.44 × 10^−5^	*LOC101103626*
15	46,934,552	0.4179	3.35 × 10^−3^	*LOC101115569*
9	84,216,970	0.4150	5.78 × 10^−3^	*TRIQK*
1	193,140,282	0.4092	1.20 × 10^−3^	*XXYLT1*
12	37,794,767	0.4068	3.08 × 10^−11^	*MROH9*
12	37,794,669	0.4068	3.90 × 10^−8^	*MROH9*
2	30,859,222	0.3954	3.36 × 10^−3^	*LOC101112450*
2	203,335,748	−0.3939	6.86 × 10^−3^	*LOC105610198*
15	44,657,493	0.3915	6.88 × 10^−3^	*OVCH2*
14	7,223,622	0.3907	6.38 × 10^−3^	*ATMIN*
14	35,898,879	0.3884	3.42 × 10^−3^	*DERPC*
18	22,586,200	−0.3828	7.77 × 10^−3^	*C18H15orf40*
9	898,695	0.3813	2.32 × 10^−8^	*LOC114116508*
19	12,755,310	0.3800	4.59 × 10^−3^	*MOBP*
1	195,315,364	0.3786	1.26 × 10^−2^	*MB21D2*
24	25,261,641	0.3768	5.31 × 10^−3^	*LOC132658555*
9	77,224,301	0.3749	8.60 × 10^−3^	*RGS22*
9	13,390,550	0.3739	5.37 × 10^−6^	*RECQL4*
12	25,015,538	0.3737	1.35 × 10^−2^	*LOC132657455*
X	36,903,853	−0.3704	8.31 × 10^−3^	*LANCL3*
17	55,374,593	0.3699	3.91 × 10^−3^	*LOC101105533*
16	32,857,591	0.3641	7.33 × 10^−3^	*OXCT1*
6	110,822,470	0.3639	3.35 × 10^−2^	*CPEB2*
14	1,033,527	0.3612	4.71 × 10^−3^	*LOC105610941*
14	16,233,790	0.3606	4.17 × 10^−2^	*LONP2*
15	42,816,579	0.3596	1.44 × 10^−2^	*LOC121816691*
7	30,447,640	0.3578	1.27 × 10^−2^	*CDIN1*
15	49,524,407	0.3559	1.67 × 10^−2^	*LOC101121373*
15	52,531,728	0.3555	9.28 × 10^−3^	*KCNE3*
4	71,238,711	0.3499	2.18 × 10^−2^	*LOC132659743*

^a^ OAA: *Ovis aries* autosome. X indicates *Ovis aries* X chromosome. ^b^ Methylation difference was calculated by subtracting the 50% NRC mean methylation from the 100% NRC mean methylation ratio.

**Table 5:** Twin-Pair Top 30 dmCpG Sites across Promoter Regions Ranked by Absolute methDiff.

OAA ^a^	Position	Mean Difference ^c^	Minimum *p*_adj_ ^b^	Gene
1	195,315,364	0.4738	1.02 × 10^−2^	*MB21D2*
18	22,586,200	−0.4717	8.35 × 10^−3^	*C18H15orf40*
12	37,794,767	0.3889	3.54 × 10^−4^	*MROH9*
12	37,794,770	0.3889	3.50 × 10^−4^	*MROH9*
11	61,786,518	0.3738	7.95 × 10^−5^	*CEP112*
11	61,786,526	0.3738	8.20 × 10^−5^	*CEP112*
11	61,786,555	0.3738	5.01 × 10^−5^	*CEP112*
11	61,786,532	0.3738	3.33 × 10^−5^	*CEP112*
24	18,537,632	0.3592	1.10 × 10^−4^	*PDILT*
24	18,537,646	0.3592	6.67 × 10^−3^	*PDILT*
24	18,537,638	0.3592	3.43 × 10^−3^	*PDILT*
16	14,514,495	0.3488	1.87 × 10^−5^	*LOC114118781*
5	35,837,678	−0.3373	1.02 × 10^−2^	*TSPAN17*
1	102,884,789	0.3233	6.57 × 10^−6^	*IVL*
1	102,884,799	0.3233	1.01 × 10^−5^	*IVL*
1	102,884,793	0.3233	6.18 × 10^−6^	*IVL*
1	102,884,829	0.3233	7.77 × 10^−5^	*IVL*
5	48,919,738	0.3218	9.16 × 10^−4^	*LOC114115044*; *LOC114115012*
5	48,919,759	0.3218	6.68 × 10^−4^	*LOC114115044*; *LOC114115012*
20	44,063,663	0.3183	2.66 × 10^−3^	*NEDD9*
4	48,749,193	0.3141	8.68 × 10^−5^	*LOC106991115*
1	217,221,696	0.3125	2.06 × 10^−5^	*EIF5A2*
15	39,955,813	0.3122	7.37 × 10^−3^	*LOC121816688*
15	39,955,801	0.3122	1.10 × 10^−2^	*LOC121816688*
15	39,955,845	0.3122	1.93 × 10^−2^	*LOC121816688*
1	102,884,928	0.3077	1.83 × 10^−3^	*IVL*
14	43,163,595	0.3040	1.26 × 10^−5^	*LOC114118137*
14	43,163,566	0.3040	5.20 × 10^−6^	*LOC114118137*
3	12,371,260	0.3010	1.85 × 10^−10^	*LOC132659379*
3	12,371,252	0.3010	4.64 × 10^−10^	*LOC132659379*

^a^ OAA: *Ovis aries* autosome. X indicates *Ovis aries* X chromosome. ^b^ Minimum *p*_adj_ ^b^ is the smallest adjusted *p*-value among the three CpGs. ^c^ Mean methDiff of the three CpGs with the largest |methDiff|.

**Table 6:** Group-Level Top 30 dmCpG Sites across Exonic Regions Ranked by Absolute methDiff.

OAA ^a^	Position	Difference ^b^	Adjusted *p*-Value	Gene
6	88,181,116	0.4847	4.30 × 10^−4^	*ADAMTS3*
11	14,521,920	0.4782	1.12 × 10^−4^	*LOC121820551*
3	211,430,308	0.4547	1.80 × 10^−4^	*C3H12orf4*
20	16,484,832	0.4537	3.26 × 10^−3^	*BICRAL*
6	34,177,841	0.4533	3.24 × 10^−4^	*CCSER1*
14	60,432,202	0.4489	1.51 × 10^−4^	*LOC105605937*
2	178,924,822	0.4444	8.50 × 10^−4^	*LOC114113196*
20	19,348,644	0.4385	1.89 × 10^−3^	*ENPP4*
2	166,471,865	0.4324	1.97 × 10^−3^	*LOC132659219*
15	76,786,768	0.4322	2.11 × 10^−3^	*PTPRJ*; *LOC101120521*
6	13,638,456	0.4304	1.30 × 10^−3^	*AP1AR*
5	12,045,802	0.4264	7.31 × 10^−4^	*LOC114114992*
3	197,427,220	0.4231	4.83 × 10^−3^	*PIK3C2G*
13	72,875,084	0.4198	5.11 × 10^−4^	*SERINC3*
3	155,626,749	0.4195	6.25 × 10^−3^	*C3H12orf56*
2	55,048,958	0.4155	8.66 × 10^−4^	*LOC121818625*
6	19,570,112	0.4147	3.13 × 10^−3^	*NPNT*
1	262,050,780	0.4119	4.23 × 10^−3^	*LOC132657453*
X	23,194,292	0.4071	8.80 × 10^−4^	*LOC114111500*
16	14,604,096	0.4071	4.04 × 10^−3^	*LOC106990331*
19	298,698	0.4040	6.76 × 10^−4^	*LOC114109393*
1	219,927,095	0.4037	2.80 × 10^−3^	*LOC132658461*
22	13,243,347	−0.4032	5.20 × 10^−4^	*FGFBP3*
11	34,236,406	0.3989	5.82 × 10^−3^	*MYO15A*
5	91,931,039	0.3985	2.05 × 10^−3^	*MCTP1*
6	101,347,205	0.3983	3.20 × 10^−3^	*ARHGAP24*
9	65,131,377	−0.3945	1.27 × 10^−2^	*CSMD3*
3	56,450,063	0.3943	5.78 × 10^−3^	*DNAH6*
20	27,195,198	0.3936	1.12 × 10^−2^	*LOC121817420*
X	13,000,445	0.3927	2.66 × 10^−3^	*LOC121818314*

^a^ OAA: *Ovis aries* autosome. X indicates *Ovis aries* X chromosome. ^b^ Methylation difference was calculated by subtracting the 50% NRC mean methylation from the 100% NRC mean methylation ratio.

**Table 7:** Twin-Pair Top 30 dmCpG Sites across Exonic Regions Ranked by Absolute methDiff.

OAA ^a^	Position	Mean Difference ^c^	Minimum *p*_adj_ ^b^	Gene
18	19,289,372	0.4743	6.34 × 10^−9^	*LOC101119079*
18	19,289,377	0.4743	6.39 × 10^−9^	*LOC101119079*
18	19,289,369	0.4743	6.39 × 10^−9^	*LOC101119079*
6	16,095,414	0.4408	2.39 × 10^−17^	*MCUB*
6	16,095,424	0.4408	3.07 × 10^−16^	*MCUB*
6	16,095,430	0.4408	5.98 × 10^−15^	*MCUB*
16	22,857,338	−0.4384	2.27 × 10^−5^	*LOC121816805*
3	95,616,014	0.4368	7.16 × 10^−7^	*DUSP11*
3	95,616,009	0.4368	7.16 × 10^−7^	*DUSP11*
14	60,505,715	0.4313	3.84 × 10^−6^	*LOC105605752*
6	17,619,562	0.4298	4.51 × 10^−4^	*LOC132659926*; *CYP2U1*; *LOC101120834*
17	17,780,552	0.4067	2.43 × 10^−3^	*MAML3*
4	46,294,679	0.4020	2.56 × 10^−4^	*RELN*
4	46,294,828	0.4020	9.37 × 10^−4^	*RELN*
3	17,175,689	0.4006	1.16 × 10^−6^	*LOC121819003*
3	17,175,703	0.4006	5.85 × 10^−6^	*LOC121819003*
3	206,750,927	0.4005	4.07 × 10^−6^	*RIMKLB*
2	2,700,060	0.3880	6.01 × 10^−5^	*LOC132659277*
2	2,700,079	0.3880	6.03 × 10^−5^	*LOC132659277*
11	8,284,215	0.3875	5.98 × 10^−4^	*LOC132657319*
11	8,284,228	0.3875	6.29 × 10^−4^	*LOC132657319*
X	133,946,776	0.3804	3.07 × 10^−9^	*GPRASP2*
X	133,946,766	0.3804	2.73 × 10^−8^	*GPRASP2*
25	8,693,487	0.3791	7.65 × 10^−4^	*LOC105604921*
14	8,050,491	0.3749	3.95 × 10^−4^	*SDR42E1*
14	8,050,526	0.3749	5.14 × 10^−4^	*SDR42E1*
14	8,050,473	0.3749	9.70 × 10^−4^	*SDR42E1*
14	60,505,728	0.3726	1.50 × 10^−3^	*LOC105605752*
20	18,260,765	0.3719	1.25 × 10^−5^	*SUPT3H*
1	173,231,278	0.3709	2.20 × 10^−3^	*BBX*

^a^ OAA: *Ovis aries* autosome. X indicates *Ovis aries* X chromosome. ^b^ Minimum *p*_adj_ ^b^ is the smallest adjusted *p*-value among the three CpGs. ^c^ Mean methDiff of the three CpGs with the largest |methDiff|.

**Table 8:** Group-Level Top 30 dmCpG Sites across Intronic Regions Ranked by Absolute methDiff.

OAA ^a^	Position	Difference ^b^	Adjusted *p*-Value	Gene
8	84,117,243	0.5598	2.55 × 10^−5^	*SLC22A2*
2	76,906,769	0.5546	9.40 × 10^−5^	*PTPRD*
16	50,813,823	0.5368	3.76 × 10^−6^	*CDH12*
2	178,059,807	0.5247	1.22 × 10^−4^	*NCKAP5*
16	61,642,271	0.5225	1.32 × 10^−4^	*CTNND2*
9	73,024,520	0.5185	8.61 × 10^−6^	*LRP12*
1	219,737,291	0.5130	2.07 × 10^−4^	*LOC101114669*
18	40,669,532	0.5042	1.11 × 10^−4^	*AKAP6*
1	194,992,857	0.5030	9.26 × 10^−5^	*PLAAT1*
4	75,164,416	−0.5001	1.04 × 10^−4^	*LOC132659718*
1	245,710,987	0.4991	1.34 × 10^−5^	*SLC9A9*
8	35,461,840	0.4906	3.53 × 10^−4^	*GRIK2*
19	5,413,691	0.4886	4.45 × 10^−5^	*GADL1*
26	10,242,265	0.4845	4.91 × 10^−6^	*TENM3*
3	90,651,461	0.4799	5.40 × 10^−5^	*LTBP1*
6	12,865,898	0.4799	2.18 × 10^−4^	*ANK2*
8	46,512,202	0.4790	1.28 × 10^−4^	*LOC132660239*
9	75,323,759	0.4788	4.75 × 10^−4^	*NCALD*
24	3,910,367	0.4782	9.54 × 10^−5^	*ADCY9*
11	14,521,920	0.4782	1.12 × 10^−4^	*LOC121820551*
17	2,744,307	0.4781	3.14 × 10^−4^	*RBM46*
3	28,900,733	0.4779	1.83 × 10^−4^	*TDRD15*
7	49,637,324	0.4776	5.53 × 10^−5^	*MINDY2*
3	97,309,839	0.4773	2.75 × 10^−4^	*GPR45*
15	69,422,459	0.4754	2.16 × 10^−2^	*LRRC4C*
15	54,029,586	0.4696	5.21 × 10^−4^	*UVRAG*
25	40,780,460	0.4694	8.58 × 10^−4^	*SHLD2*
6	97,125,811	0.4675	1.13 × 10^−4^	*RASGEF1B*
2	85,905,644	0.4669	2.08 × 10^−4^	*CNTLN*
1	80,267,384	0.4663	2.93 × 10^−4^	*COL11A1*

^a^ OAA: *Ovis aries* autosome. X indicates *Ovis aries* X chromosome. ^b^ Methylation difference was calculated by subtracting the 50% NRC mean methylation from the 100% NRC mean methylation ratio.

**Table 9:** Twin-Pair Top 30 dmCpG Sites across Intronic Regions Ranked by Absolute methDiff.

OAA ^a^	Position	Mean Difference ^c^	Minimum *p*_adj_ ^b^	Gene
2	20,201,463	0.6573	3.92 × 10^−3^	*CYLC2*
X	5,299,121	0.6039	3.10 × 10^−9^	*LOC132658770*
14	1,825,665	0.5671	1.08 × 10^−3^	*WDR59*
3	85,170,517	0.5279	3.49 × 10^−22^	*SOS1*
19	37,983,264	0.5098	7.51 × 10^−19^	*SYNPR*
19	37,983,276	0.5098	2.62 × 10^−16^	*SYNPR*
5	66,798,240	0.4959	3.06 × 10^−9^	*ADAM19*
5	66,798,222	0.4959	8.91 × 10^−9^	*ADAM19*
21	47,375,553	0.4950	4.52 × 10^−5^	*LOC114110140*
2	190,034,994	0.4894	7.45 × 10^−6^	*CNTNAP5*
10	27,172,802	0.4854	3.22 × 10^−12^	*LOC114116606*
10	27,172,805	0.4854	2.49 × 10^−7^	*LOC114116606*
10	27,172,796	0.4854	2.49 × 10^−7^	*LOC114116606*
13	43,651,862	0.4785	2.07 × 10^−10^	*LOC105611101*
9	6,888,609	0.4708	2.34 × 10^−7^	*LOC101103735*
9	6,888,641	0.4708	3.52 × 10^−6^	*LOC101103735*
10	28,191,192	0.4690	8.24 × 10^−5^	*STARD13*
10	28,191,203	0.4690	1.49 × 10^−4^	*STARD13*
2	95,713,462	0.4676	4.98 × 10^−4^	*IFT74*
2	59,952,502	0.4653	1.80 × 10^−7^	*PRUNE2*
1	245,710,987	0.4642	1.17 × 10^−4^	*SLC9A9*
21	47,375,534	0.4583	4.32 × 10^−6^	*LOC114110140*
2	88,276,064	0.4535	2.25 × 10^−6^	*SLC24A2*
2	198,240,538	0.4529	1.09 × 10^−12^	*DNAH7*
13	34,489,997	0.4511	2.85 × 10^−6^	*JCAD*
13	34,490,002	0.4511	3.92 × 10^−7^	*JCAD*
5	55,693,105	0.4509	1.18 × 10^−3^	*PPP2R2B*
1	87,278,576	0.4496	3.58 × 10^−10^	*LOC132658075*
1	87,278,568	0.4496	7.33 × 10^−10^	*LOC132658075*
1	87,278,556	0.4496	7.33 × 10^−10^	*LOC132658075*

^a^ OAA: *Ovis aries* autosome. X indicates *Ovis aries* X chromosome. ^b^ Minimum *p*_adj_ ^b^ is the smallest adjusted *p*-value among the three CpGs. ^c^ Mean methDiff of the three CpGs with the largest |methDiff|.

## Data Availability

The original data presented in the study are openly available in Synapse at https://doi.org/10.7303/syn72086670.1.

## Supplementary Materials

The following supporting information can be downloaded at: https://www.mdpi.com/article/10.3390/ijms27031553/s1.
