# Supplementary material for: Maternal Nutrient Restriction Programs Fetal Hepatic DNA Methylation in Ovine Monozygotic Twins
Source: Int J Mol Sci. 2026 Feb 4;27(3):1553. doi: 10.3390/ijms27031553 (PMC12897638; doi:10.3390/ijms27031553)
Supplement: Supplementary file 1 [file ijms-27-01553-s001.zip › ijms-4104161-supplementary.pdf]

Supplemental Table S1. Whole-Genome Bisulfite Sequencing Quality Control Metrics.

Groups	Sample ID	# Reads	Uniquely Aligned (%)	% BS Conversion (Non-CpG)	% BS Conversion (Spike-in)	Unique CpG	Avg. CpG Coverage
50% NRC	Pair_1_50	546,433,574	76.8%	99.3%	99.5%	49,849,696	11X
	Pair_2_50	520,562,934	77.2%	99.4%	99.5%	49,427,929	11X
	Pair_3_50	567,022,609	77.1%	99.4%	99.5%	49,873,324	12X
	Pair_4_50	471,800,573	76.9%	99.4%	99.5%	49,498,288	10X
100% NRC	Pair_1_100	591,902,364	76.9%	99.4%	99.5%	50,319,655	12X
	Pair_2_100	525,236,132	77.4%	99.4%	99.5%	50,197,378	11X
	Pair_3_100	600,723,232	75.7%	99.4%	99.5%	49,206,261	12X
	Pair_4_100	538,604,754	77.4%	99.4%	99.5%	49,489,561	11X

Supplemental Table S2. Summary of Differentially Methylated CpG (dmCpG) Sites.

	Total dmCpGs	Positive methDiff [a]	Negative methDiff [b]	Promoter	Exon	Intron	Genic	Intergenic
**Panel A:** Group-Level File (Treatment-level Comparison; padj ≤ 0.05; ∣methDiff∣ ≥ 0.10)								
50 vs 100	1,636,305	1,614,260	22,045	40,533	126,667	785,381	830,632	783,214
**Panel B:** Twin-Pair Files (Pairwise Comparisons; padj ≤ 0.05; ∣methDiff∣ ≥ 0.10)								
Pair 1	5,292,038	5,213,557	78,481	154,941	581,219	2,758,644	2,960,409	2,255,747
Pair 2	1,695,156	1,617,706	77,450	49,708	190,630	854,049	922,257	747,308
Pair 3	557,579	454,245	103,334	18,363	71,463	282,192	308,730	240,046
Pair 4	271,624	108,405	163,219	9,421	35,352	140,577	153,201	113,849

[a] Indicates increased methylation in 50% NRC relative to 100% NRC (i.e., hypermethylation in restricted [RES] group)
[b] Indicates decreased methylation in 50% NRC relative to 100% NRC (i.e., hypomethylation in restricted [RES] group)
Note: Sites may appear in multiple categories due to overlapping annotated features.

Supplemental Table S3. Liver-Sparing Effect across Monozygotic Twin Pairs.

Comparison	Diet	Fetal Weight	Liver Weight	Liver g/kg	LSI [a]
Pair 1	RES	4.636	108.57	23.4	0.791
	CON	5.810	171.91	29.6	
Pair 2	RES	4.326	107.62	24.9	0.820
	CON	4.610	139.85	30.3	
Pair 3	RES	5.532	132.72	24.0	0.900
	CON	5.750	153.24	26.7	
Pair 4	RES	5.328	128.09	24.0	1.048
	CON	6.240	143.11	23.0	

[a]LSI = (liver g/kg) $_{RES}$ / (liver g/kg) $_{CON}$; >1 indicates relative liver sparing; <1 indicates relative liver hit.

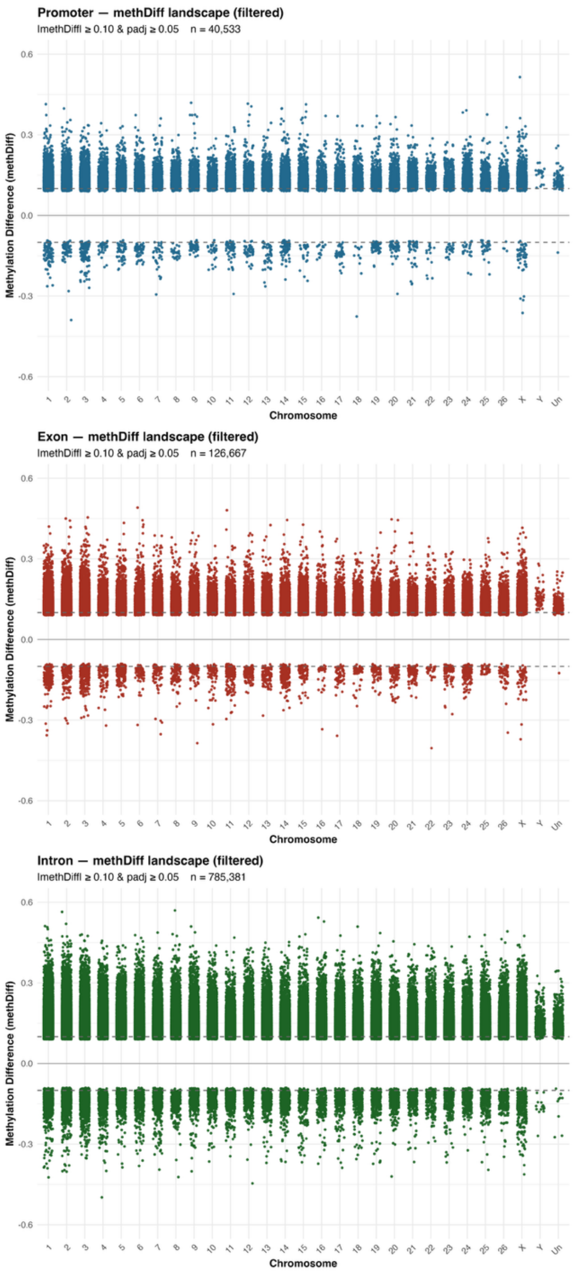

Supplemental Figure S1. Chromosomal distribution and effect size (methDiff) of dmCpGs across genomic features within the Group-Level Analyses. The x-axis represents the chromosome number, and the y-axis shows the methylation difference. Each point represents the |methDiff| value associated with a differentially methylated site in the Group-Level Analysis.

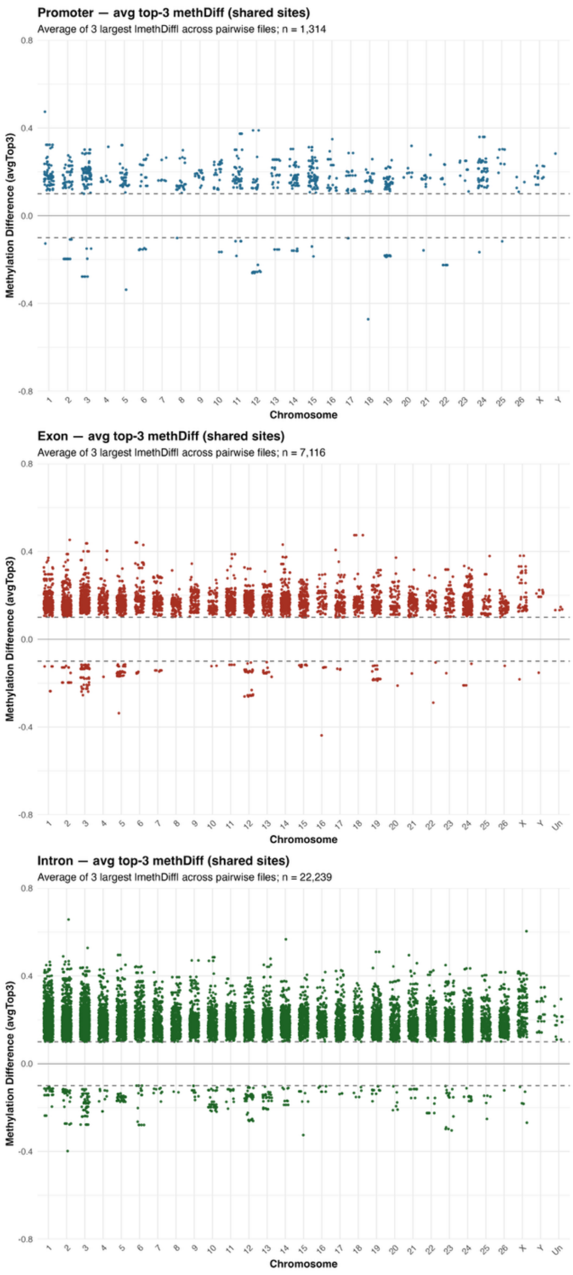

Supplemental Figure S2. Chromosomal distribution and effect size (methDiff) of dmCpGs across genomic features within the Twin-Pair Analyses. The x-axis represents the chromosome number, and the y-axis shows the methylation difference. Each point represents an average of the three largest |methDiff| values across pairwise files.

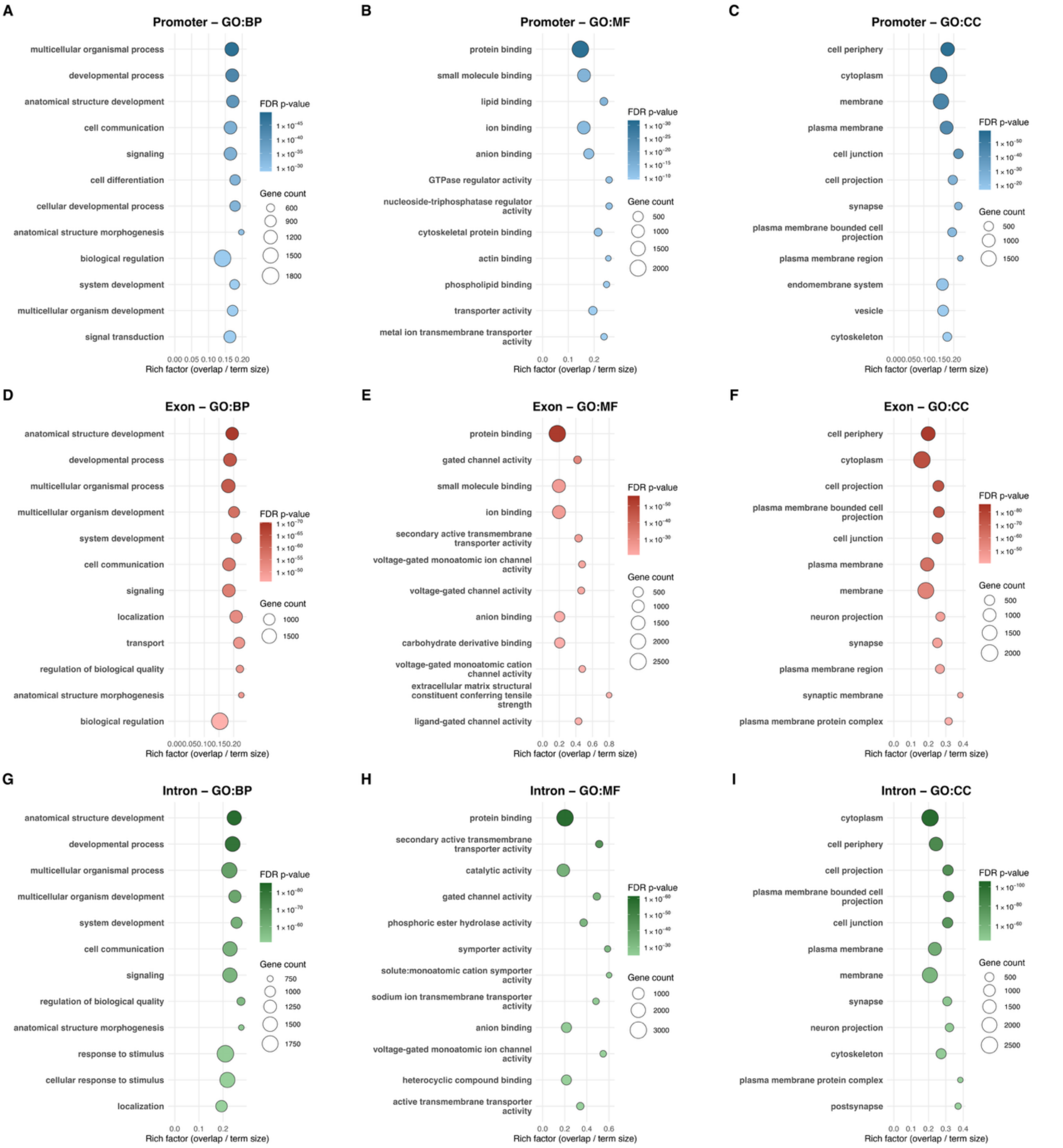

Supplemental Figure S3. Group-Level Gene Ontology enrichment for promoter-, exon-, and intron-associated dmCpGs across Biological Process (BP), Molecular Function (MF), and Cellular Component (CC) categories in the Group-Level Analyses. Panels A–C show GO:BP, GO:MF, and GO:CC enrichments for promoter-associated genes. Panels D–F display corresponding GO enrichments for exon-associated genes. Panels G–I illustrate GO enrichments for intron-associated genes. The x-axis represents the rich factor (overlap/term size), and the y-axis lists pathway names. Point size reflects the number of dmCpG-associated genes, and color indicates the adjusted p-value (FDR q ≤ 0.05).

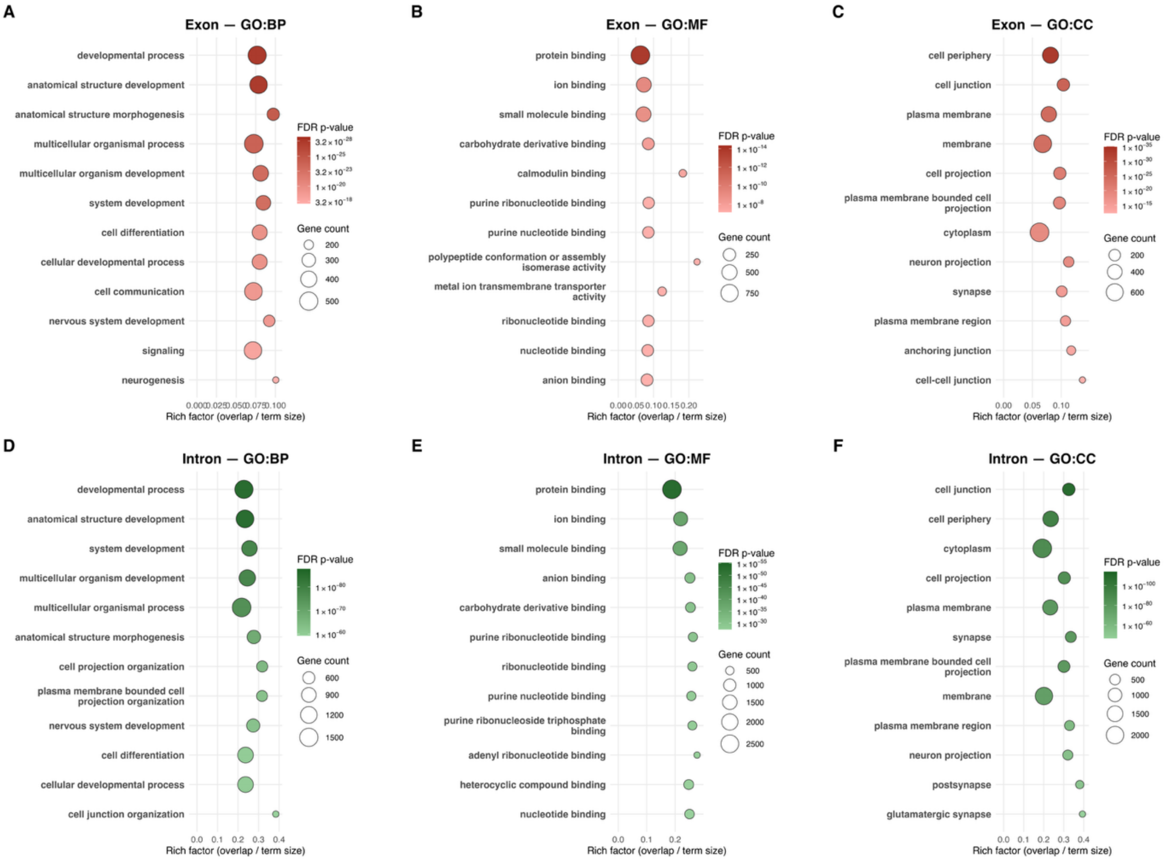

Supplemental Figure S4. Twin-Pair Gene Ontology enrichment for exon- and intron-associated dmCpGs across Biological Process (BP), Molecular Function (MF), and Cellular Component (CC) categories in the Twin-Pair Analyses. Panels A–C show GO:BP, GO:MF, and GO:CC enrichments for exon-associated genes. Panels D–F illustrate GO enrichments for intron-associated genes. The x-axis represents the rich factor (overlap/term size), and the y-axis lists pathway names. Point size reflects the number of dmCpG-associated genes, and color indicates the adjusted p-value (FDR q ≤ 0.05).
